# A Remedy for the Vomitings of Early Pregnant Women

**Published:** 1844-03-23

**Authors:** 

**Affiliations:** Baden


A REMEDY FOR THE VOMITINGS OF EARLY PREGNANT WOMEN. BY PITSCHAFT, OF BADEN.

M. Pitschaft says he has discovered a remedy for the distressing vomitings which generally so much affect women in the early stages of pregnancy. His remedy consists of pills of creosote, henbane powder, and distilled water, one to be taken three times a day.—*Ibid from Ibid.*

